# Would the one-stage combined approach lead to better long-term neurological outcomes than the posterior approach alone in multilevel degenerative cervical myelopathy patients with T2-Weighted increased signal intensity? An 8-year follow-up results and propensity score matching analysis

**DOI:** 10.1186/s12891-024-07554-3

**Published:** 2024-06-06

**Authors:** Ruomu Qu, Ben Wang, Yiyuan Yang, Zexiang Liu, Zhuo Chen, Yunxia Wu, Xiumao Li, Liang Jiang, Xiaoguang Liu, Zhongjun Liu

**Affiliations:** 1 Orthopaedic Department, Peking University Third Hospital, Beijing, China; 2 Engineering Research Center of Bone and Joint Precision Medicine, Peking University, Beijing, China; 3 Beijing Key Laboratory of Spinal Disease Research, Peking University, Beijing, China; 4 Peking University Health and Science Center, Beijing, China; 5 Department of Orthopedic Surgery, the Second Affiliated Hospital, Zhejiang University School of Medicine, Hangzhou City, Zhejiang Province PR China

**Keywords:** One-stage combined approach, Laminoplasty, Degenerative cervical myelopathy, Surgical outcome

## Abstract

**Background:**

T2-weighted increased signal intensity (ISI) is commonly recognized as a sign of more severe spinal cord lesions, usually accompanied by worse neurological deficits and possibly worse postoperative neurological recovery. The combined approach could achieve better decompression and better neurological recovery for multilevel degenerative cervical myelopathy (MDCM). The choice of surgical approach for MDCM with intramedullary T2-weighted ISI remains disputed. This study aimed to compare the neurological outcomes of posterior and one-stage combined posteroanterior approaches for MDCM with T2-weighted ISI.

**Methods:**

A total of 83 consecutive MDCM patients with confirmed ISI with at least three intervertebral segments operated between 2012 and 2014 were retrospectively enrolled. Preoperative demographic, radiological and clinical condition variables were collected, and neurological conditions were evaluated by the Japanese Orthopedic Assessment score (JOA) and Neck Disability Index (NDI). Propensity score matching analysis was conducted to produce pairs of patients with comparable preoperative conditions from the posterior-alone and combined groups. Both short-term and mid-term surgical outcomes were evaluated, including the JOA recovery rate (JOARR), NDI improvements, complications, and reoperations.

**Results:**

A total of 83 patients were enrolled, of which 38 and 45 patients underwent posterior surgery alone and one-stage posteroanterior surgery, respectively. After propensity score matching, 38 pairs of comparable patients from the posterior and combined groups were matched. The matched groups presented similar preoperative clinical and radiological features and the mean follow-up duration were 111.6 ± 8.9 months. The preoperative JOA scores of the posterior and combined groups were 11.5 ± 2.2 and 11.1 ± 2.3, respectively (*p* = 0.613). The combined group presented with prolonged surgery duration(108.8 ± 28.0 and 186.1 ± 47.3 min, *p* = 0.028) and greater blood loss(276.3 ± 139.1 and 382.1 ± 283.1 ml, *p*<0.001). At short-term follow-up, the combined group presented a higher JOARR than the posterior group (posterior group: 50.7%±46.6%, combined group: 70.4%±20.3%, *p* = 0.024), while no significant difference in JOARR was observed between the groups at long-term follow-up (posterior group: 49.2%±48.5%, combined group: 59.6%±47.6%, *p* = 0.136). No significant difference was found in the overall complication and reoperation rates.

**Conclusions:**

For MDCM patients with ISI, both posterior and one-stage posteroanterior approaches could achieve considerable neurological alleviations in short-term and long-term follow-up. With greater surgical trauma, the combined group presented better short-term JOARR but did not show higher efficacy in long-term neurological function preservation in patients with comparable preoperative conditions.

## Background

Degenerative cervical myelopathy (DCM) is one of the most common causes of progressive neurological dysfunction and disability in older individuals worldwide and can result in severe neurological deficits, such as loss of extremity dexterity, gait instability and sensory deficits [1–4]. Magnetic resonance imaging (MRI) can assess the severity of DCM and reveal cord compression and intramedullary signal intensity [5, 6]. T2-weighted increased signal intensity (ISI) is commonly recognized as a sign of more severe spinal cord lesions and is usually associated with worse neurological deficits and adverse postoperative neurological recovery [7–12].

With multilevel (three or more intervertebral segments) involvement, patients with multilevel degenerative cervical myelopathy (MDCM) usually present with more severe stenosis, cord compression, neurological deficits and a higher incidence of ISI [13–15]. For MDCM patients with ISI, the patient outcome recovery could be unsatisfactory. It is of great clinical relevance to clarify the choice of surgical approach in MDCM patients with ISI to maximize neurologic recovery [16, 17].

Three surgical approaches are conventionally used for MDCM: anterior, posterior, and combined approaches [18, 19]. The anterior approach applied to multilevel anterior compressions, such as disc herniation, osteophytes and non-severe ossification of posterior longitudinal ligament (OPLL); the posterior approach could applied to multilevel canal stenosis, ossification of ligamentum flavum and severe OPLL; the one-stage combined posteroanterior approach, which offered circumferential decompression was a reserved option for severe MDCM patients with multilevel canal stenosis and severe ventral compression [20–23]. However, whether the combined approach could achieve better outcomes than the posterior approach alone in MCDM patients with ISI remains controversial. Some surgeons believed that it was a more appropriate approach for MDCM patients with ISI, while some surgeons suggested that the neurological deterioration in the cord where presenting ISI is irreversible, and the posterior and combined approach would result in similar neurological outcomes; thus, the posterior approach alone is sufficient.

Using the propensity score matching method, this study aimed to compare the short-term and long-term clinical outcomes of the one-stage combined posteroanterior approach and posterior approach alone for MDCM patients with ISI. Our hypothesis is that the one-stage posteroanterior approach might maintain better neurological recovery over a longer duration in MDCM patients with T2-weighted ISI.

## Methods

### Patient population

Data from consecutive patients who underwent surgery for confirmed DCM between January 2012 and March 2014 at our institute were retrospectively collected. The medical records, surgical characteristics, and imaging features were systematically reviewed. Patients who underwent cervical surgery with confirmed MDCM were enrolled based on the following strict inclusion criteria: (1) radiographically confirmed DCM with clinical symptoms, signs, and confirmed intramedullary ISI on T2-weighted MRI [24]. (2) at least three surgical segments were operated on via the posterior-alone or combined posteroanterior approach; and (3) a minimum of 96 months of follow-up [25, 26]. The exclusion criteria were as follows: (1) surgery involving a spinal tumor or trauma, (2) cervical kyphosis > 10° (C2-7 Cobb angle), (3) accompanying thoracic or lumbar diseases, (4) any previous cervical spinal surgery history [27] and (5) accompanying ossification of longitudinal ligament (OPLL).

### Surgical management

All surgeries were performed by the same experienced surgical team. The posterior approach used multilevel open-door laminoplasty; the one-stage combined approach consisted of an initial multilevel open-door laminoplasty in the prone position (posterior stage) and a subsequent single-level ACDF at the site that presented the brightest ISI in the supine position as described (anterior stage). The choice of surgical approach was based on spinal cord compression characteristics and decided by our spine surgical team discussion, but the ultimate choice of procedure was decided upon by the patient before the operation agreement was signed [22]. The one-stage posteroanterior combined approach was usually suggested in patients presenting with multilevel spinal canal stenosis and severe ventral compression, whereas the posterior-alone approach was employed in patients presenting with multilevel spinal canal stenosis with or without severe ventral compression. Postoperatively, patients were advised to wear a soft cervical collar for comfort in the following 4 weeks.

### Radiological assessments

**Fig. 1:**
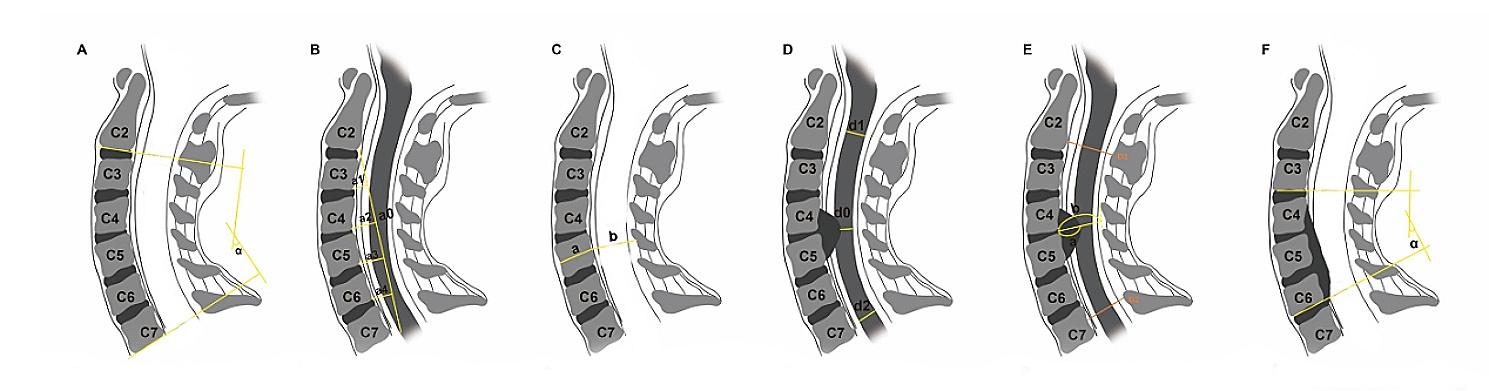
Sagittal measurements. **A**: C2-C7 Cobb angle = α; **B**: CI=(a1 + a2 + a3 + a4)/a0 × 100%; **C**: Pavlov ratio = b/a; **D**: SSCC = 2*d0/(d1 + d2); **E**: Sagittal occupancy ratio = a/b; SCC = 2*(b-a)/(D1 + D2); **F**: focal Cobb angle = α

**Fig. 2:**
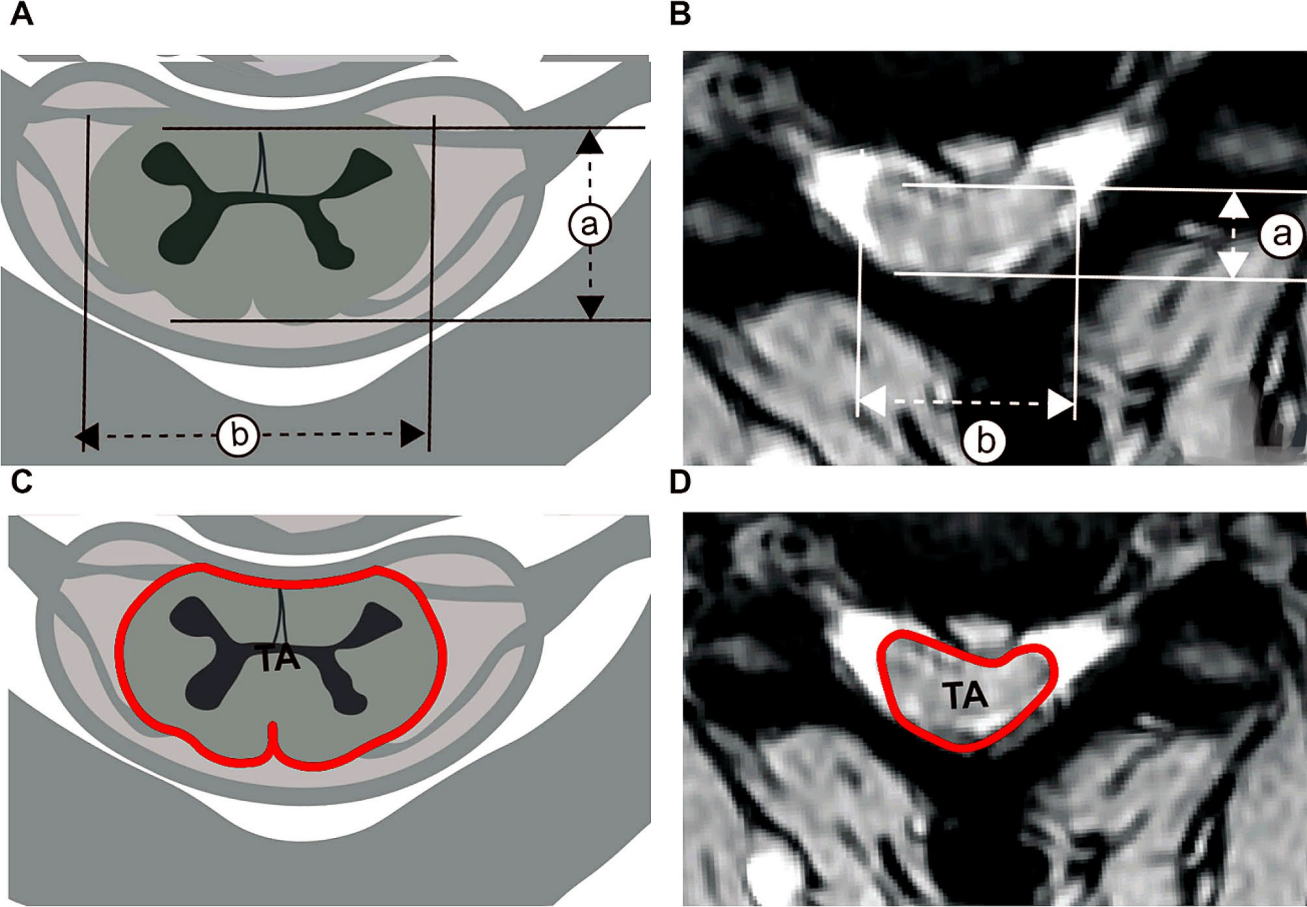
Transverse measurements. TA: transverse area of cord which was circled by red line, AP: anteroposterior diameter = a, TCC: transverse cord compression = a/b

The preoperative radiological examinations were reviewed, including lateral radiography, computed tomography (CT), and magnetic resonance imaging (MRI). Cervical alignment was evaluated in neutral lateral radiographs using the C2-7 Cobb angle, focal Cobb angle (cobb angle of the involved segments) and Ishihara’s curvature index (CI) [17]. The average Pavlov ratio at levels C3 through C6 was calculated to evaluate the developmental cervical spinal stenosis [28]. At the segment with maximal cord compression on T2-weighted magnetic resonance images (T2WI), we assessed the anteroposterior (AP) diameter [29] and transverse area (TA) of the spinal cord in the transverse measurements (Fig. 1); and the sagittal occupancy ratio of the canal [30], sagittal spinal cord compression (SSCC) and sagittal canal compromise (SCC) in the sagittal measurements (Fig. 2). The measurements of ISI included maximum vertical length and relative intensity (RI) of ISI [13]. The RI was defined as the grayscale of the ISI region divided by that of the spinal cord at the C1 level: RI = (grayscale of ISI)/(grayscale at the cord at the C1 level). The maximum vertical length of the ISI was also measured on sagittal T2WI [13]. All measurements were performed twice by trained orthopedic residents.

### Outcome assessments

The clinical outcomes were assessed at two follow-up points postoperatively: short- (over 12 months) and long-term (over 96 months). The neurological outcomes of the patients were assessed using the Japanese Orthopaedic Association (JOA) score [31] and neck disability index (NDI). The JOA recovery rate (JOARR) was calculated according to the Hirabayashi method: JOARR = (postoperative JOA score–preoperative JOA score)/(17 [full score]–preoperative JOA score) × 100% [32]. All patients were classified into four recovery grades based on the JOARR: JOARR < 25% as poor, 25%~50% as fair, 50%~75% JOARR as good, and 75% < JOARR as excellent [33]. The rates of excellent and good recovery were calculated.

Perioperative complications were assessed using medical records, including cerebrospinal fluid (CSF) leakage, wound complications, hematoma, esophageal fistula, C5 palsy, and iatrogenic neurological injury. Residual complications were evaluated at the final follow-up, including dysphagia, dysphonia, C5 palsy, axial symptoms, and reoperations. Postoperative axial symptoms were identified as any complaint of discomfort involving the neck, shoulder, or periscapular region, including pain, fatigue, stiffness, and tightness [16].

### Statistical analysis

SAS (version 9.2; SAS Institute Inc., Cary, NC) was employed for the statistical analysis. Categorical variables are expressed as percentages and counts, and continuous variables are expressed as the mean value ± standard deviation. T tests, chi square tests, ANOVA and nonparametric analysis were employed to compare the differences between surgical approaches [23]. Propensity score matching analysis was applied in our study to adjust for confounding biases. The propensity score of the surgical approach was first calculated with the C2-7 Cobb angle and sagittal occupancy ratio in a logistic regression model. The C-statistic, which suggests fitting, was 0.61, implying fair efficacy. The patients in the posterior group and one-stage posteroanterior group were matched according to calculated propensity scores on the condition that the caliper was lower than 0.1. Statistical significance was considered when the two-tailed p value was less than 0.05.

## Results

### Patient characteristics

A total of 676 patients who underwent cervical surgeries that were performed by our spinal surgery team between January 2012 and March 2014 were retrospectively reviewed. Based on the inclusion and exclusion criteria, 83 MDCM patients with T2-weighted intramedullary ISI were included, of which 38 patients underwent posterior surgery and 45 patients underwent one-stage posteroanterior combined surgery.

The medical records and radiological characteristics were systematically investigated. The propensity score of the surgical approach was first calculated with the C2-7 Cobb angle and sagittal occupancy ratio in a logistic regression model. The C-statistic, which suggests fitting, was 0.61, implying fair efficacy. The patients in the posterior group and one-stage posteroanterior group were matched according to calculated propensity scores on the condition that the caliper was lower than 0.1.

After propensity score matching analysis, 38 pairs of comparable patients from the one-stage posteroanterior group and posterior group were matched. The average follow-up duration was 111.6 ± 8.9 months. Both groups did not present significant differences in demographic data, symptom duration or accompanying diseases (Table 1). The average total follow-up periods of the posterior group and combined group were 109.8 ± 9.4 months and 113.3 ± 8.0 months (*p* = 0.081), respectively. Both groups shared similar preoperative JOA scores and NDI scores.

**Table 1:** Baseline demographic and radiological characteristics in the posterior and one-stage posteroanterior groups before and after propensity score matching

	Before Propensity Score Matching			After Propensity Score Matching		
	Posterior Group (n = 38)	Combined Group (n = 45)	P value	Posterior Group (n = 38)	Combined Group (n = 38)	P value
Age(yr)	56.5 ± 9.7	54.6 ± 9.1	0.507	56.47 ± 9.70	54.71 ± 9.13	0.560
Male sex	31(81.6%)	30(66.7%)	0.127	31(81.6%)	24(63.2%)	0.074
BMI	25.5 ± 3.3	25.4 ± 3.5	0.809	25.5 ± 3.3	25.1 ± 3.6	0.503
Diabetes Mellitus	2(5.3%)	7(15.6%)	0.135	2(5.3%)	7(18.4%)	0.078
Hypertension	12(31.6%)	15(33.3%)	0.866	12(31.6%)	13(34.2%)	0.808
Drinker	6(15.8%)	6(13.3%)	0.753	6(15.8%)	6(15.8%)	1.000
Smoker	9(23.7%)	12(26.7%)	0.757	9(23.7%)	10(26.3%)	0.792
Symptom duration	49.7 ± 75.2	49.9 ± 65.5	0.480	49.7 ± 75.2	46.6 ± 63.5	0.688
Preoperative NDI	7.1 ± 7.8	7.2 ± 6.4	0.704	7.1 ± 7.9	7.4 ± 6.6	0.635
Preoperative JOA	11.5 ± 2.2	11.1 ± 2.3	0.592	11.5 ± 2.2	11.1 ± 2.3	0.613

The results are presented as the mean ± standard deviation, number (percentage), or number only

*Abbreviations* BMI: body mass index; JOA: Japanese Orthopaedics Association Score; NDI: neck disability index

### Preoperative radiological measurements

Both groups did not show significant differences in overall preoperative radiological features. The patients in both groups showed similar and comparable cord compression and cervical lordosis (Table 2).

**Table 2:** Preoperative radiological measurements

Variable	Before Propensity Score Matching			After Propensity Score Matching		
	Posterior Group (n = 38)	Combined Group (n = 45)	P value	Posterior Group (n = 38)	Combined Group (n = 38)	P value
Average Pavlov ratio	74.4%±10.3%	73.0%±11.2%	0.459	74.4%±10.3%	73.1%±11.6%	0.500
AP (mm)	3.3 ± 1.4	3.7 ± 1.1	0.157	3.3 ± 1.4	3.7 ± 1.1	0.180
TA (mm^2^)	48.7 ± 17.5	49.4 ± 13.9	0.565	48.7 ± 17.5	48.9 ± 13.7	0.747
TCC	20.7%±9.2%	23.5%±8.0%	0.157	20.7%±9.2%	23.7%±8.1%	0.158
Sagittal occupancy ratio of the canal	47.1%±13.6%	50.4%±9.8%	0.281	47.1%±13.6%	49.4%±9.8%	0.467
SCC	30.1%±10.9%	30.5%±8.6%	0.281	30.1%±10.9%	30.7%±8.7%	0.618
SSCC	53.5%±18.0%	52.4%±15.9%	0.805	53.5%±18.0%	53.0%±16.3%	0.917
CI	6.2 ± 11.0	7.1 ± 12.2	0.942	6.2 ± 11.0	7.2 ± 12.1	0.934
Length of ISI (mm)	8.0 ± 4.7	7.7 ± 4.2	0.869	8.0 ± 4.7	7.7 ± 4.5	0.795
ISI relative intensity	2.16 ± 0.58	2.18 ± 0.73	0.891	2.16 ± 0.58	2.18 ± 0.69	0.975
C2-C7 Cobb Angle	8.7 ± 11.0	8.5 ± 12.0	0.674	8.7 ± 11.0	9.2 ± 12.8	0.901
Focal Cobb Angle	6.1 ± 10.1	5.6 ± 11.0	0.708	6.1 ± 10.1	6.2 ± 11.3	0.926

The results are presented as the mean ± standard deviation, number (percentage), or number only

*Abbreviations* AP: anteroposterior diameter of the spinal cord; TA: transverse area of the spinal cord; TCC: transverse cord compression; SCC: sagittal cord compromise; SSCC: sagittal spinal cord compression; CI: Ishihara curvature index; ISI: increased signal intensity

### Operation and complication characteristics

Both the patients in the posterior and one-stage posteroanterior groups had 5.1 ± 0.2 segments operated on. The one-stage posteroanterior group had a longer surgery duration (186.1 ± 47.3 min and 108.8 ± 28.0 min, respectively, *p* = 0.028) and more blood loss than the posterior alone group (382.1 ± 283.1 mL and 276.3 ± 139.1 ml, respectively, *p* < 0.001). The average hospital stays of patients in the posterior and combined groups were 8.9 ± 3.0 and 9.9 ± 4.0 days (*p* = 0.284), respectively, and the postoperative hospital stays were 5.9 ± 2.0 and 6.5 ± 2.2 days, respectively (*p* = 0.203).

Both groups did not differ significantly in the overall complication rate. No severe perioperative complications, such as death, paralysis, esophageal fistula, hematoma, or dysphonia, were observed in either group. There were two cases of CSF leakage, one case of C5 palsy, and one case of wound complication reported in the one-stage posteroanterior group and two cases of wound complication and one case of C5 palsy in the posterior group. At the final follow-up, two patients (5.3%) in the one-stage posteroanterior group reported dysphagia (*p* = 0.155). Additionally, thirteen patients (34.2%) in the posterior group and fourteen (36.8%) patients in the one-stage posteroanterior group reported remaining axial symptoms (*p* = 0.812). One (2.6%) patient in the posterior group underwent reoperation because of recurrent symptoms, while no reoperation was observed in the one-stage posteroanterior group (*p* = 0.317) (Table 3).

**Table 3:** Operation-related characteristics and complications

Variable	Posterior group (n = 38)	Combined group (n = 38)	P value
Segments operated on	5.1 ± 0.2	5.1 ± 0.2	1
Blood Loss(ml)	276.3 ± 139.1	382.1 ± 283.1	<0.001
Surgery Duration(mins)	108.8 ± 28.0	186.1 ± 47.3	0.028
Hospital Stay(days)	8.9 ± 3.0	9.9 ± 4.0	0.284
Postoperative Hospital stays	5.9 ± 2.0	6.5 ± 2.2	0.203
Dysphagia	0	2(5.3%)	0.155
Wound complication	2(5.3%)	1(2.6%)	0.558
CSF leakage	0	2(5.3%)	0.155
Axial Symptoms	13(34.2%)	14(36.8%)	0.812
C5 Palsy	1(2.6%)	1(2.6%)	1
Reoperation	1(2.6%)	0	0.317

The results are presented as the mean ± standard deviation, number (percentage), or number only

*Abbreviations* CSF: cerebral spinal fluid

### Outcome assessment

At the short-term follow-up, both the posterior and combined groups showed considerably improved postoperative JOA scores (14.7 ± 1.5 and 15.2 ± 1.6, *p* = 0.071) (Table 4). The JOA improvements were 3.3 ± 2.1 and 4.1 ± 2.0 (*p* = 0.180). The JOARRs of the posterior and one-stage posteroanterior groups were 50.7%±46.6% and 70.4%±20.3%, respectively (*p* = 0.024). The one-stage combined posteroanterior group presented a significantly higher JOARR than the posterior group.

At the long-term follow-up, both groups maintained favorable postoperative JOA scores (14.8 ± 1.6 and 14.9 ± 2.4, *p* = 0.232). The long-term JOA improvements were 3.6 ± 2.7 and 3.7 ± 3.2 (*p* = 0.864). The JOARRs of the posterior and one-stage posteroanterior groups were 49.2%±48.5% and 59.6%±47.6%, respectively (*p* = 0.136).

**Table 4:** Clinical outcomes at short-term and long-term follow-up in the posterior and combined groups

	Posterior group (n = 38)	Combined group (n = 38)	P value
Short-term follow-up duration (months)	21.0 ± 7.9	24.2 ± 6.7	0.052
Short-term postoperative JOA	14.7 ± 1.5	15.2 ± 1.6	0.071
Short-term JOA improvement	3.3 ± 2.1	4.1 ± 2.0	0.180
Short-term JOARR	50.7%±46.6%	70.4%±20.3%	0.024
Short-term Postoperative NDI	1.3 ± 2.8	1.8 ± 3.1	0.203
Short-term NDI improvement	5.9 ± 8.0	5.6 ± 7.3	1.000
Long-term follow-up duration (months)	109.8 ± 9.4	113.3 ± 8.0	0.081
Long-term postoperative JOA	14.8 ± 1.6	14.9 ± 2.4	0.232
Long-term JOA improvement	3.6 ± 2.7	3.7 ± 3.2	0.864
Long-term JOARR	49.2%±48.5%	59.6%±47.6%	0.136
Long-term Postoperative NDI	1.3 ± 2.6	1.8 ± 3.1	0.320
Long-term NDI improvement	5.8 ± 7.8	5.6 ± 7.3	0.979

The results are presented as the mean ± standard deviation, number (percentage), or number only

*Abbreviations* JOA: Japanese Orthopaedics Association score; NDI: Neck Disability Index

## Discussion

The choice of ideal decompression strategy of MCDM patients with T2WI ISI is challenging in clinical practice. Though the one-stage combined approach would theoretically provide more thorough decompression compared to posterior laminoplasty alone, few study has compared the long-term efficacy of the combined approach and posterior approach alone. This study aimed to investigate whether the one-stage combined posteroanterior approach could lead to better neurological outcomes than posterior approach alone for MDCM patients with T2-weighted ISI.

### T2-weighted increased signal intensity

Intramedullary ISI on T2-weighted MRI was first reported by Takahashi as a result of chronic mechanical compression and venous infarction [8, 34]. Recognized as an important sign in the preoperative evaluation and prognosis prediction of DCM, as severe ISI could be related to severe cord lesions and less recuperative potential, while milder ISI may be related to milder cord alterations and higher recuperative potential [5, 6]. Yukawa et al. [15] categorized sagittal T2-weighted ISI into 3 grades and associated a higher grade of ISI with a worse neurological outcome. Although an increasing number of studies have revealed that patients presenting with preoperative ISI might have a worse neurologic outcome following surgery, few studies have investigated the appropriate surgical strategy for patients with preoperative intramedullary T2-weighted ISI on MRI to improve neurological outcomes [5, 35].

### The treatment approach for MDCM

The optimal surgical treatment for MDCM remains disputed. These patients usually present with multilevel stenosis, severe cord compression and significant neurological deficits. The conventional surgical approaches used for MDCM were the anterior approach (anterior cervical discectomy and fusion [ACDF] and anterior cervical corpectomy and fusion [ACCF]) and the posterior approach (laminoplasty or laminectomy with fusion). Ghogawala Z et al. compared the anterior and posterior approaches in a randomized clinical trial and reported the both approaches in similar efficacy in improving 1-year patient-reported physical function [36]. For patients presenting with severe ventral compression, some surgeons have suggested a one-stage combined posteroanterior approach to acquire sufficient decompression. Studies has compared the short-term efficacy of combined approach and the posterior approach [21–23], however there results were not consistent and few studies have compared the one-stage combined approach and posterior approach for MDCM patients with intramedullary ISI, especially for long-term neurological recovery. From our experience, the combined approach might provide sufficient decompression and anterior fixation and consequently possible better neurological recovery. Hence, we conducted this study, and our hypothesis was that the combined approach might maintain higher neurological recovery than the posterior approach alone in the long-term follow-up.

### Handling preoperative heterogeneity and analysis of the results

The preoperative heterogeneity among patients between groups has led to difficulty in comparing the efficacy of different approaches. In this study, propensity score matching analysis was conducted to ensure the preoperative homogeneity of patients from both groups and thereby reducing selection bias. After PSM, the matched cases showed similar preoperative clinical conditions and radiological features, making the comparison between groups reasonable. Regarding short-term outcomes, the combined group showed higher postoperative JOA scores and JOA improvements, and significantly higher he JOARR than the posterior group. Regarding the long-term outcome, no significant difference was observed in postoperative JOA, JOA improvements and JOARRs between the combined group and posterior groups. The possible explanations for this phenomenon might be as follows: The patients with ISI have more severe cord lesions and lower recuperative potential. At the short-term follow-up, the combined posteroanterior approach could achieve more sufficient decompression that leads to higher neurological function recovery, while this effect is not as strong as in patients without ISI due to the limit of recuperative potential. At the long-term follow-up, with aging and possible re-degeneration, the overall physical function of patient deteriorated [37], and this superiority in JOARR became no longer significant to be observed. Additional investigation involving postoperative radiological follow-up is required to clarify the possible mechanisms.

### Safety and complications

Safety and possible perioperative complications are also major concerns influencing surgical planning in clinical practice. In this study, the one-stage posteroanterior combined approach resulted in more blood loss and prolonged surgery duration than the posterior approach. Although no significant difference was found in the overall complication rate, two cases of CSF leakage and two cases of dysphagia were reported in the one-stage posteroanterior group, while no such case was found in the posterior approach group. This result was consistent with Fehlings et al. [38], who reported that patients in the combined approach showed an increased risk of postoperative complications, especially dysphagia, and Li [22], who described comparable overall complication rates of the combined approach. Additionally, none of the patients in the one-stage posteroanterior group underwent reoperation, whereas one patient in the posterior group received additional surgery (ACDF) due to recurrent symptoms.

### Limitations

Our study has several limitations. First, this was a single-center retrospective study, and conclusions were drawn based on single-center data. Further multicenter prospective studies with a larger sample size and continuous follow-up will make it possible to further investigate neurological recovery over time. Second, there was still measurement bias in the radiological evaluation. Third, though we have reduced the preoperative heterogeneity of patients in the combined group and posterior group by propensity score matching, the patients in both groups may present different pathologies, which introduced selection bias and restrict the accessibility of our results. Additionally, investigations involving long-term postoperative radiographic characteristics, radiomics and machine learning methods might be able to clarify the possible mechanisms [39].

## Conclusions

For MDCM patients with ISI, both posterior and one-stage posteroanterior approaches could achieve considerable neurological alleviations in short-term and long-term follow-up. The combined approach presented with prolonged surgery duration, greater blood loss and a higher incidence of dysphagia and CSF leakage. Although the combined approach showed better short-term neurological function recovery than the posterior approach, no such superiority was found in long-term follow-up.

## Data Availability

The datasets used and/or analyzed during the current study are available from the corresponding author upon reasonable request.

## Abbreviations

DCM: Degenerative cervical myelopathy
MDCM: Multilevel degenerative cervicalmyelopathy
JOA: Japanese Orthopedic Assessment score
JOARR: Japanese Orthopedic Assessment score recovery rate
ISI: Increased signal intensity
ACDF: Anterior cervical discectomy and fusion
ACCF: Anterior cervical corpectomy and fusion
CSF: Cerebrospinal fluid
CT: Computed tomography
MRI: Magnetic resonance imaging
T2WI: T2-weighted magnetic resonance images
DM: Diabetes mellitus
NDI: Neck disability index
AP: Anteroposterior diameter of the spinal cord
TA: Transverse area of the spinal cord
SCC: Sagittal cord compromise
SSCC: Sagittal spinal cord compression
CI: Ishihara curvature index
OPLL: Ossification of posterior longitudinal ligament
OLF: Ossification of ligamentum flavum
